# Clinical and Functional Outcomes of Ilizarov Bone Transport in Traumatic Tibial Bone Loss

**DOI:** 10.5704/MOJ.2507.012

**Published:** 2025-07

**Authors:** N Mohd-Yusof, RY Kow, AF Sulong, RM Nallayan, MA Zainal-Abidin

**Affiliations:** 1 Department of Orthopaedics, Traumatology and Rehabilitation, International Islamic University Malaysia, Kuantan, Malaysia; 2 Department of Community Medicine, International Islamic University Malaysia, Kuantan, Malaysia

**Keywords:** external fixator, osteogenesis, distraction, tibial fractures, bone loss

## Abstract

**Introduction:**

Bone loss is a serious complication following an open fracture or fracture-related infection in the tibia. Treatment with Ilizarov bone transport in this condition is preferred because it minimises additional soft tissue injury and is able to close the bone and soft tissue defects through distraction osteogenesis. The objective of this study is to evaluate the relationship between functional outcomes of patients with tibial bone loss treated with Ilizarov bone transport and return to work.

**Materials and methods:**

A cross-sectional study among 40 patients was carried out in 2 public hospitals. Patient records and radiographs were reviewed for information on the initial injuries, treatment, union of bone, and complications while on treatment. The clinical outcomes were evaluated with ASAMI Bone grading system while the functional outcome of the affected limb was assessed using Lower Extremity Score (LEFS) and ASAMI Functional grading system at least 10 months after the removal of the Ilizarov external fixator.

**Results:**

Thirty-eight (95%) achieved union. Thirty-six (90.0%) patients had excellent and good scores for clinical and functional results, respectively using the ASAMI grading system. The mean LEFS is 80.1% (range 58 to 91%). Thirty-three (82.5%) patients were able to return to work. The clinical outcome has a strong and positive correlation with functional outcome both on ASAMI functional score and LEFS (p<0.001). Patients with good and excellent ASAMI functional scores significantly correlate with higher odds to return to work (p<0.001). Return to work was also associated with a higher LEFS score (p=0.006).

**Conclusion:**

Most patients with tibial non-union treated with Ilizarov bone transport have good and excellent clinical and functional outcomes and are able to return to work. Return to work significantly correlates with good functional outcomes.

## Introduction

Fracture of the tibia is more commonly associated with open fracture, vascular injuries and compartment syndrome than any other bone. These conditions are risk factors for infection, delayed union, mal-union and non-union. Tibial bone loss often results from initial trauma, debridement or infection. Ilizarov external fixator is an ideal implant to treat bone defects of the tibia because it is able to close the wound and bone gradually through distraction osteogenesis. It reduces the need for complex free tissue transfer procedures and donor site morbidity from bone grafts. Although the Ilizarov external fixator is associated with complications such as pain, pin site infection and joint stiffness, it is usually resolved after removing the fixator^1-5^. Many studies have been done to look at the clinical and functional outcomes following bone transport at the tibia. However, its correlation to return to work remains unexplored.

Therefore, the aim of this study is to evaluate the clinical and functional outcome of patients treated for traumatic bone loss of the tibia with Ilizarov bone transport and its correlation to return to work.

## Materials and Methods

This is a cross-sectional study of patients who underwent Ilizarov bone transport to treat tibial bone loss from October 2015 to October 2019 at Hospital Tengku Ampuan Afzan (HTAA), Kuantan, Pahang and Hospital Tuanku Ja'afar (HTJ), Seremban. Excluded from this study are those who are less than 18 years old, involving intra-articular fragments, and those who had other associated injuries.

Aktuglu *et al* found that following bone transport, the excellent and good rates in bone and functional results are 83.3% and 95.8%, respectively^6^. Based on the study, the proportion was estimated at 0.90 and the degree of precision was taken to be 10%. A sample size of 40 patients was included in this study considering 10% non-response rate. The study was approved by the Ethics Review Board of both institutions (NMRR-18-3861-44477).

Patients' demographics and clinical data including age, gender, surgical indications, organisms isolated, consolidation time, time for external fixation and bone transport, external fixation index, any observed complications and follow-up duration were gathered through a review of the medical record.

Fifty-four medical records of patients with traumatic tibial defects treated with Ilizarov fixators were identified during the study period. Fourteen were excluded because unable to be contacted for the assessment of the functional outcome at least 10 months after the removal of the external fixator. Forty patients fulfilled the criteria at the end of the study. There were 32 males and 8 females. The mean age group is 32.2 years old (range 18 – 49 years old).

**Fig. 1:**
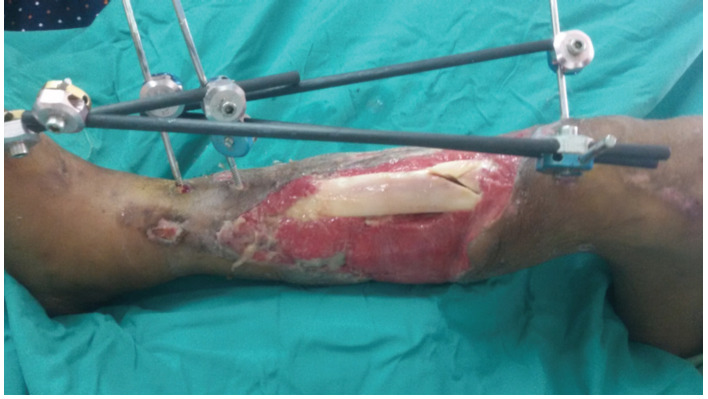
Clinical photograph of a grade IIIB open tibial fracture stabilized with an external fixator.

Thirty-six were due to road traffic accidents, two falls from height, and one each from heavy metal fall over leg and assault injury. Thirty-six cases had initially open fractures while four cases were closed. Twelve cases occur at the proximal tibia while 14 cases each at the midshaft and distal tibia. Fig. 1 depicts a 33-year-old man with a grade IIIB open tibial fracture following a road traffic accident.

**Fig. 2:**
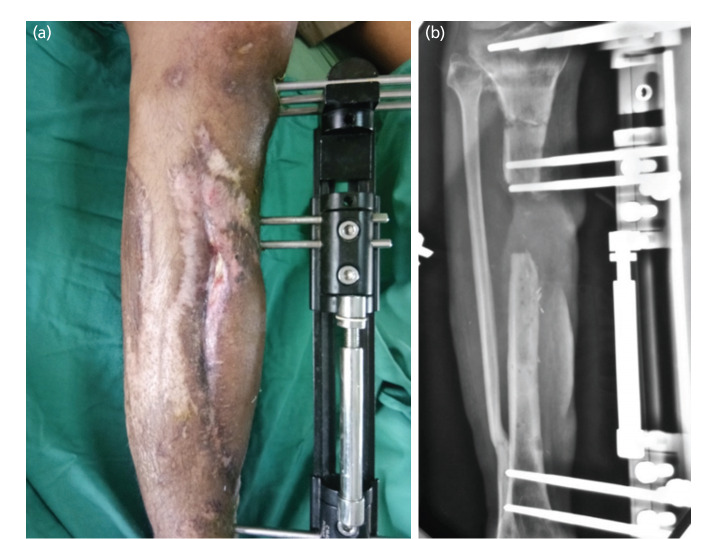
(a) Soft tissue defect corrected by anterolateral thigh flap. (b) Image showing the use of a monorail external fixator.

The initial treatments were external fixator in 14 patients, intramedullary nailing in 12 patients, plating in 7 patients, limb reconstruction system (LRS) and cast application in 1 patient, respectively. Fig. 2 demonstrates the initial outcomes after debridement, anterolateral thigh flap, and monorail external fixator. The mean bone defect was 3.9cm (range 28). The majority of patients had a 3cm (42.5%) bone defect. Two patients had the longest bone defect of 8cm following an open fracture. With regards to wound coverage, 25 (62.5%) of the participants had Split Skin Graft (SSG) or tissue flap whereas 15 (37.5%) patients had none. Out of 25 patients who had SSG or flap, 11 patients had SSG alone. Thirteen patients had local flaps for coverage of the tibial wound: six had gastrocnemius flap, four had a soleus flap, two had a perforator flap and one patient had an anterolateral thigh flap (ALT) and tibialis anterior flap each.

**Fig. 3:**
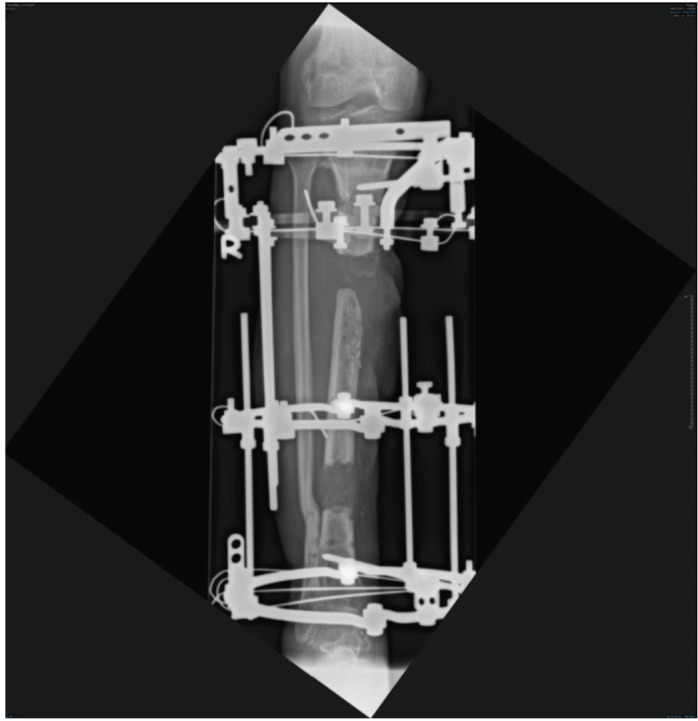
Ilizarov external fixator used to bridge the bone defect.

**Fig. 4:**
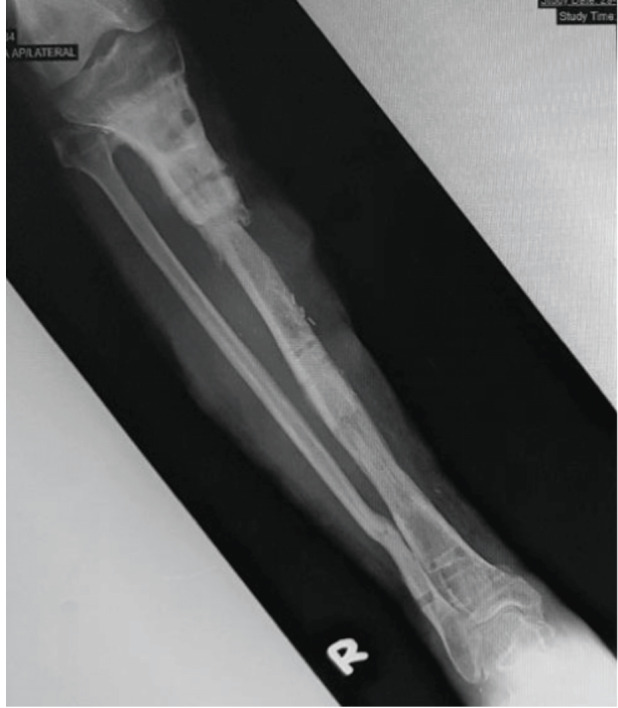
Radiograph showing bone defect consolidation 12 months after Ilizarov external fixator application.

Twenty-one (52.5%) had a history of infection before the bone transport procedure. In the presence of infection, the infected area is debrided and stabilised with an external fixator. Intravenous antibiotics are given based on the microbiological result. Bone transport is done after the infection has been controlled. Fig. 3 shows a retrograde bifocal bone transport was done one year and three months after the initial injury to bridge the bone defect using the Ilizarov external fixator. Fig. 4, the patient was able to return to work after the bone consolidated 12 months after application of Ilizarov external fixator.

For surgical procedures and post-operative rehabilitation, the Ilizarov external fixation is applied with transosseous wires and Schanz pins. The first wire is inserted parallel to the knee joint. Then, an Ilizarov fixator is mounted, ensuring it is aligned with the tibia. Once the frame has been completely attached to the bone and the proximal and distal fragments of the tibiae are aligned, a corticotomy is made at the metaphyseal area. Bone transport is started after a seven-day latency period with the rate of quarter turn four times a day. Once the transport segment has reached the distal segment, the docking site is open, and the interposing tissue is cleared. An autologous bone graft was then added around the bone end to enhance union.

The patients were followed up every two weeks during the bone transport and every six weeks during the consolidation phase. During the follow-up, the patients were checked for any complications such as pin site infection and joint stiffness. Physiotherapy with weight bearing as tolerated is encouraged at this time. The Ilizarov external fixation was removed after four cortices of regenerate had ossified on the anterior-posterior and lateral plain radiograph. The plain radiographs were also reviewed to assess the alignment and bony union of the tibia.

The bone (clinical) and functional results were evaluated using Association for the Study and Application of the Method of Ilizarov (ASAMI) classification. Bone (clinical) results were evaluated by four criteria: union, infection, deformity and limb-length discrepancy. Functional results were evaluated by five criteria: active, limp, minimum stiffness of knee or ankle joint (loss of more than 15° of full extension of the knee or 15° of dorsiflexion of the ankle in comparison with the normal contralateral ankle), reflex sympathetic dystrophy and pain^7^.

Overall functional assessment of the limb was done after 10 months of removal of the Ilizarov ring using a validated Bahasa Malaysia version of the Lower Extremity Functional Scale (M-LEFS) questionnaire developed by Mohd Yunus *et al* from the Binkley English Version of Lower Extremity Functional Scale (LEFS)^8^.

The limb function was assessed using M-LEFS points. Patients gave points, 0 – 4, to the activity listed in the questionnaire. Point 0- extreme difficulty to perform activity, 1- quite a bit of difficulty, 2- moderate difficulty, 3- a little bit of difficulty, and 4- no difficulty. The Malay version of LEFS is a patient self-reported assessment with 20 questions related to their daily activities, such as sitting, walking, climbing stairs, and standing, with a maximum score of 80. Return to work was classified as a return to full duties, a return to light duties, or unable to work.

Data was collected, entered and cleaned in Microsoft Excel software before being analysed using IBM SPSS software Version 29.0. Data was presented descriptively using count and percentages for categorical data, mean and standard deviation and range for continuous data. Fisher’s exact test and Mann-Whitney U test was used to find the association between categorical and continuous factor with a return-to-work variable. The crude odds ratio was calculated to estimate the odds for return to work and presented with 95% confidence interval based on Altman. Kendall’s Tau-B correlation test was done to find the correlation between ASAMI Category and LEFS Score with return to work. All tests were 2-sided, and alpha was set at 0.05.

## Results

Thirty-eight (95%) patients achieved union with a mean union time of 11.6 months (range 7-24 months). The mean external fixator time is 11.5 months with a mean external fixator index (EFI) of 3.16 months/cm (range 2 to 6 months/cm). EFI is defined as the duration of external fixation in months divided by the total amount of bone transported in centimetres. Thirty-six (90%) patients had excellent and good ASAMI bone and functional results (Table I and II). The median LEFS score is 64 (80 % of maximal function) ranging from 46 to 73 (58 to 91 % of maximal function).

**Table I:** Distribution of ASAMI Bone (clinical) result.

ASAMI Bone Result	Frequency	Percentage
Excellent	23	57.5%
Good	13	32.5%
Fair	2	5.0%
Poor	2	5.0%
Total	40	100.0%

**Table II:** Distribution of ASAMI functional result.

ASAMI Functional Result	Frequency	Percentage
Excellent	13	32.5%
Good	23	57.5%
Fair	1	2.5%
Poor	3	7.5%
Total	40	100.0%

No intraoperative complication was observed during the procedure. However, post-operative complication was encountered in 65% of our cases during the treatment. The most common complication was a pin-track infection, which occurred in 12 (30%) patients. Ten patients had only local inflammation, which was treated by pin care after a swab for culture sensitivity was taken. The inflammation subsided with regular dressings and antibiotics. The remaining two patients who had pin loosening associated with purulent discharge underwent pin removal, wound debridement and reapplication of pins (Table III).

**Table III:** List of complications.

Complication	Frequency (%)
Pin tract infection	12 (30.0%)
Surgical site infection	6 (15%)
Limb length discrepancy	3 (7.5%)
Knee stiffness	9 (22.5%)
Ankle stiffness	8 (20.0%)
Knee contracture	2 (5.0%)
Ankle equines	2 (5.0%)

Surgical site infection noted in six patients were managed by debridement. Pus and devitalised tissues were sent for culture and sensitivity. Eight patient specimens grew Staphylococcus aureus, four grew Proteus sp, three patients grew two organisms Pseudomonas and Escherichia coli, two grew methicillin-resistant Staphylococcus aureus and one grew Coagulase-negative Staphylococcus. An appropriate intravenous antibiotic was started according to the sensitivity of the organism. All infections were resolved before the end of treatment.

Knee and ankle stiffness were seen in nine and eight patients, respectively. Three of our patients had obvious limps due to limb length discrepancy (LLD). The LLD was in the range of 3.5 – 4.5cm. They were not keen on further surgical intervention and were put on modified footwear. Five of our patients had LLD less than 2.0cm which was not troubling them pursuing their daily activity or causing obvious deformity of the limb. Knee contracture and ankle equinus were seen in two patients each. All patients with knee and ankle stiffness subsequently improved with regular physiotherapy. All patients had pain during the distraction period and the pain was well controlled by adequate analgesics. There were no limb-threatening complications such as neurovascular injury and compartment syndrome. In this study, none required amputation including the two patients with non-union.

In terms of employment, 33 (82.5%) patients went back to their jobs. Twenty-one patients (52.5%) were performing full duty whereas 12 (30.0%) patients were doing light duty. Only 7 (17.5%) patients were unable to work. The main reason was difficulty to fully weight bear and their injury had restricted them from their jobs.

Using Kendall’s tau-b test, there is a strong and positive correlation between ASAMI Bone (clinical) and ASAMI functional outcomes, τb=0.724, P<0.001 and the LEFS outcome τb=0.435, P<.001 (Table IV).

**Table IV:** Relationship between functional outcome and return to work (n=40).

Functional Outcome	Not Working, n (%)	Working, n (%)	Odd Ratio	P Value
ASAMI Category				<0.001*
Poor and Fair	4 (57.1)	0 (0)	Baseline	
Good and Excellent	3 (42.9)	33 (100)	88 (3.7, 2092)	
LEFS Score, Median IQR)	50.0 (13.0)	65.0 (8.0)	-	<0.001**

Notes - *Fisher’s Exact test, **Mann-Whitney U test. Return to work outcome was binarily categorised into working (light duty and full duty) and not working. While ASAMI functional outcome categorically into two. Being good and excellent ASAMI outcome correlated with higher odds of working outcome. Working outcome was also significantly associated with higher mean LEFS Score.

Return to work outcome was binarily categorised into working (light-duty and full duty) and not working. While ASAMI functional outcome is categorically into two. Being good and excellent ASAMI outcome correlated with higher odds of working outcome (Table V).

**Table V:** Correlation between outcomes with ASAMI bone functional outcomes (n=40).

Functional Outcome	n	τb	95% CI	P Value
ASAMI Category	40	0.724	(0.604, 0.812)	<0.001*
LEFS Score, Mean (SD)	40	0.435	(0.245, 0.593)	<0.001*

Notes - *p statistically significant at <0.05. Correlation between ASAMI Bone Clinical result with ASAMI bone functional outcome and LEFS Score. Using Kendall’s tau-b test, there is strong and positive correlation between ASAMI Bone clinical and ASAMI functional outcomes, τb =0.724, P<0.001 and the LEFS score outcome τb =0.435, P<0.001.

**Fig. 5:**
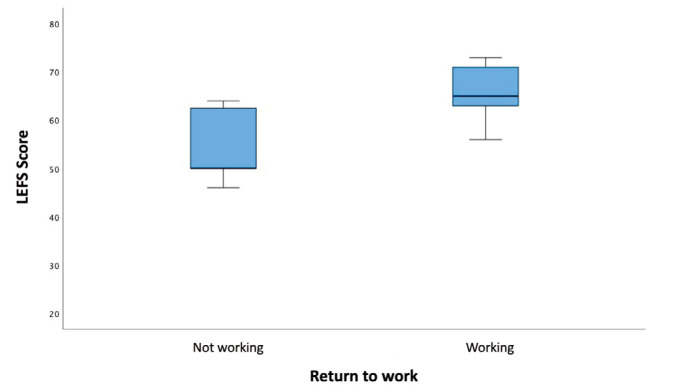
LEFS score and return to work. The median and interquartile range (IQR) for the non-working and working groups are 50.0 (13.0) and 65.0 (8.0), respectively.

Fig. 5 shows the LEFS score in the group of patients who had returned to work and the group of patients who had not returned to work.

## Discussion

In our study of 40 patients, we had 42 complications at a rate of 1.05 complications per patient which was lesser compared to other studies. Yin *et al* in their systematic review and meta-analysis of Ilizarov methods for the treatment of infectious femur and tibia stated an average complication per patient with infected tibia non-union was 1.231. In another systemic review by Aktuglu *et al* on Ilizarov bone transport for tibia non-union which included twenty-seven articles with 619 patients, the mean complication rate per patient was 1.229.

Pin-track infection was identified as being the most common complication in both studies. In our study, there were 12 (30.0%) cases of pin tract infection manifested as pain, erythema and small purulent discharge around the pin sites.

Non-compliance to pin site dressing leads to a high rate of pin tract infection, as most of our patients stay far from the hospital. These patients were managed by pin site dressing, local wound care and administration of the appropriate antibiotic. Daily pin site care plays an important role in the treatment of pin site infections^10^. Pin site infection usually occurs in areas where there is a greater range of motion and high stress. A recent study by Ceroni *et al* suggested that excessive movement at the fixator pin-bone interface leads to pin-site irritation and infection^11^. Although superficial pin-tract infections were observed in many of our patients, no cases of deep infection were reported. Similar results were shown in a study conducted by Xu *et al* in which 100% of the patient’s achieved union while none of the patients developed a deep infection as a complication of using the Ilizarov technique for infected non-unions of the tibia^12^.

We had 6 (15.0%) cases of surgical site infection which were confined to the surgical scar and subcutaneous tissue. These infected wounds were successfully debrided.

Another common problem to be tackled is joint stiffness. Use of a ring fixator near the joint results in joint stiffness, with subsequent development of dysfunction and joint space narrowing. The reported frequency is around 25%^13^. In our study, 9 (22.5%) patients were unable to flex their knee fully, whereas another 8 (20.0%) patients showed loss of ankle dorsiflexion of more than 5° after removal of the frames. After regular physiotherapy for more than 3 months, 70% of them gained a full range of movements in both joints. Two patients had fixed equinus deformities and they were not keen for further treatment.

There was no re-fracture, malunion of the tibia or amputation surgery performed for failed Ilizarov treatment at our centres. According to Yin *et al*, malunion was seen in 7% and limb amputation in 4% of the patients treated with the Ilizarov method^14^. In contrast, Papakostidis *et al* reported 5% re-fracture and 2.9% amputation in their study^15^.

Many of our patients (82.5%) were able to return to work with no amputation or persistent infection. Liodakis *et al* reported 62% of their patients were able to return to work^16^. Six (15.4%) of their patients underwent amputation because of infection. We had 90% good to excellent clinical and functional outcomes which are similar to other results. Eralp *et al* reported 91.9% good to excellent clinical and 90.5% excellent to good functional results^17^.

Our rate of return to work is similar to study done in the European countries. Dendrinos *et al* in Greece had a similar rate of return to work although their clinical and functional outcomes were lesser^4^. While Corona *et al*^5^ in Spain reported a better clinical and functional outcome but with a similar rate of return to work (Table VI).

**Table VI:** Clinical, functional outcome and return to work.

Authors (years)	Number of patients	ASAMI good and excellent Bone Scores (%)	ASAMI good and excellent Functional Scores (%)	Return to Work (%)
Dendrinos *et al*^4^	28	79	67	82
Corona *et al*^5^	31	100	86	83
Present study	40	90	90	83

## Conclusion

Clinical outcome correlates with good functional outcome. Patients with good and excellent functional scores significantly correlate with higher odds of returning to work.
